# Global Diversity of Norovirus Genotypes from 2002 to 2025: A Review of Temporal, Age-Specific and Regional Trends

**DOI:** 10.3390/v18101083

**Published:** 2026-10-01

**Authors:** Jennifer L. Cannon, Christine Kim, Duncan Mahood, Brooke A. Bollman, Guha Asthagiri Arunkumar, Benjamin A. Lopman, Katherine B. Carlson

**Affiliations:** 1 Epidemiologic Research & Methods, LLC, Southport, NC 28641, USA; jennifer.cannon@epidemiologymethods.com (J.L.C.);; 2 Moderna, Inc., Cambridge, MA 02142, USA; 3 Rollins School of Public Health, Emory University, Atlanta, GA 30322, USA

**Keywords:** norovirus, genotype diversity, molecular epidemiology, acute gastroenteritis, sporadic infection, global surveillance, vaccine

## Abstract

Norovirus is a leading cause of acute gastroenteritis (AGE) worldwide, with significant morbidity and mortality, particularly in young children and older adults. This study characterizes the global prevalence and diversity of norovirus genotypes among sporadic cases. A review of English-language studies (published 2002–2026) that characterized norovirus genotype distribution among sporadic cases was conducted, excluding outbreak and asymptomatic detections. Pooled data were analyzed with stratifications by age group, region, and year of sample collection. A total of 20,588 cases were identified from 72 publications. There were 30 genotypes identified, with GII.4, GII.3, GII.2, GII.17, and GII.6 and each accounting for ≥5% of detections. GII.4 was the predominant genotype globally, accounting for 54% of cases and up to 80% among older adults across all years, but periodic fluctuation was seen, coinciding with fluctuations in the prevalence of other genotypes, especially GII.3, GII.2, and GII.17. Among GI genotypes, GI.3 was most prevalent (46%), particularly among adults. Genotype diversity was highest among children in low- and middle-income countries and in Asia/Oceania. Findings indicate that a small number of genotypes account for most cases. As genotyping characterizes the major capsid protein, the primary target of antibody recognition, findings have important implications for vaccine development and public health interventions.

## 1. Introduction

Norovirus is a leading cause of acute gastroenteritis (AGE) worldwide, responsible for an estimated 677 million illnesses annually [1]. Although illness is typically considered self-limiting in healthy individuals, norovirus infection can cause severe disease, particularly in young children, older adults, and individuals with underlying medical conditions [2,3,4]. Large norovirus outbreaks with high attack rates frequently occur in closed and semi-closed settings, including hospitals, long-term care facilities, schools, childcare centers, cruise ships and catered events; however, sporadic infections are substantially less well characterized because of limited routine surveillance and diagnostic testing [5]. The global economic impact of norovirus gastroenteritis is also substantial, with estimated annual global costs of $4.2 billion in direct healthcare expenses and $60.3 billion in societal losses [1,6].

Noroviruses are genetically diverse and are classified into multiple genogroups and genotypes based on the amino acid sequence of the major capsid protein VP1, the major antigenic target of infection- and vaccine-induced antibodies [7]. Although several genogroups can infect humans, the vast majority of human norovirus disease is caused by viruses belonging to genogroups GI and GII [8]. Over the past two decades, genogroup II, genotype 4 (GII.4) has been the most frequently detected genotype globally [9,10,11,12], although most available data were derived from outbreak studies, particularly for adult populations [13,14]. GII.4 predominance is characterized by the periodic emergence of new GII.4 variants which historically have replaced prior GII.4 variants [15]. The substantial genetic and antigenic diversity of noroviruses, together with the continued evolution of GII.4 viruses and others, complicates understanding of immune protection, as the degree to which prior infection confers cross-protection against different genotypes or antigenically distinct strains within a genotype remains an active area of investigation [15,16].

This study presents a comprehensive analysis of the global norovirus genotype prevalence among sporadic cases across diverse age groups and geographic regions from 2002 to 2025. Unlike much of the existing literature, which is derived from outbreak investigations and may disproportionately capture genotypes associated with large-scale transmission events, this analysis focuses on sporadic infections that more accurately reflect the broader burden of norovirus disease in the community. By synthesizing data from published studies, we characterize patterns of genotype distribution and diversity across populations and over time. These findings provide critical insight into the molecular epidemiology of human noroviruses and offer an evidence base to support design of multivalent vaccines and guide public health interventions.

## 2. Methods

### 2.1. Data Sources and Extraction

We conducted a comprehensive review of English-language literature (published 2002–2026) to identify studies reporting norovirus genotype data from individual, sporadic cases. Searches were conducted in PubMed and EMBASE (Ovid) and included a combination of the relevant subject headings and text words to represent human norovirus and genotypes (Supplemental Text). All records were downloaded into EndNote and de-duplicated. Searches were conducted on 15 March 2023 (1901 records) and updated on 13 May 2024 (139 new records), 29 May 2025 (114 new records) and 7 May 2026 (99 new records). The included studies: (1) reported genotype data for genogroup I and II noroviruses, (2) provided age range (Tables S1 and S2) of included cases and/or geographic region, and (3) provided data from sporadic AGE cases that could be extracted from the text, tables or figures. WebPlotDigitizer v5.2 [17] was used to aid in data extraction from figures. Studies reporting only outbreak data were excluded. Data from sporadic cases were excluded if combined with data from outbreaks or asymptomatic subjects as reported. Data were extracted by one researcher (DM) and independently verified by a second (JLC). Discrepancies were resolved through discussion or by a third reviewer, if necessary. Studies were screened by researchers for redundant reporting in multiple studies. The total number of cases attributed to each norovirus genotype was extracted and categorized by age group, geographic region, and year of sample collection (Table S1).

### 2.2. Data Analysis

Prevalence estimates for each norovirus genotype were calculated by summing the total number of cases attributed to each genotype and dividing by the total number of cases with successful genotyping data. Analyses were stratified by age group, geographic region, or year of sample collection. Studies spanning two partial calendar years were assigned a norovirus season based on peak norovirus activity (e.g., a study conducted September 2002 to August 2003 in North America was assigned to 2002/2003, reflecting a winter seasonality in the Northern Hemisphere). For studies reporting only aggregate data spanning 2 to 5 years, the midpoint norovirus season was used, excluding studies aggregating over 5 years of data and using the first full season for studies aggregating 2 years of data. We used the age categories defined in the text. Although the category ranges overlapped, each case was assigned to a single category to ensure mutually exclusive grouping for analysis as shown in Tables S1 and S2. Geographic regions were defined using the United Nations geoscheme [18], consolidated into five categories (North America, Central and South America, Europe, Africa and Asia/Oceania) due to small sample size in some subregions.

## 3. Results

### 3.1. Overall Distribution of Genotypes Among Norovirus Cases

**Figure 1:**
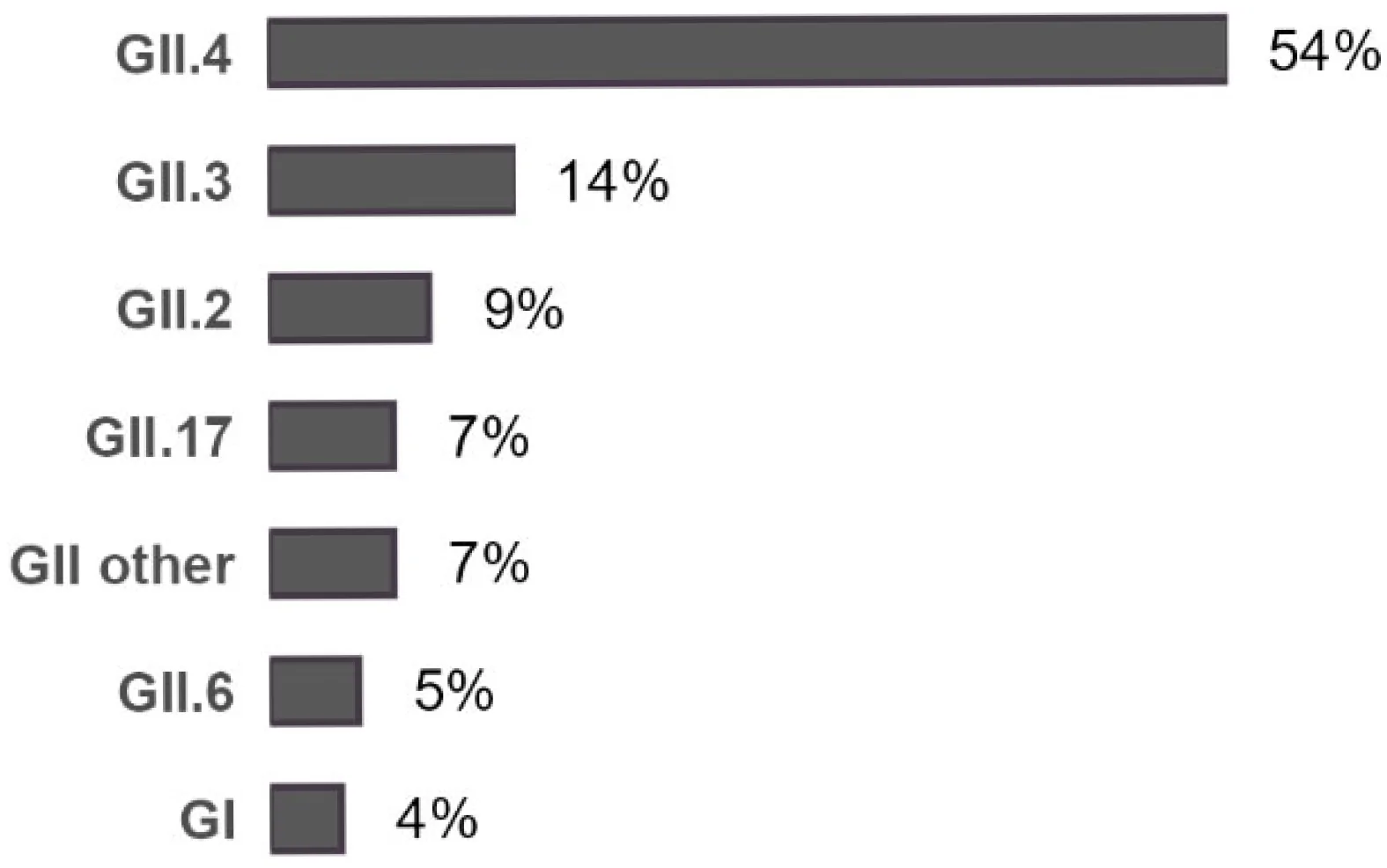
Overall distribution of genotypes among norovirus cases (*n* = 20,588) from the 2002/2003 to 2024/2025 norovirus seasons. Overall distribution of genotypes among norovirus cases (*n* = 20,588) from the 2002/2003 to 2024/2025 norovirus seasons.

A total of 72 studies were included in the analysis (Table S1), representing *n* = 20,588 individual norovirus cases with genotype data between the 2002/2003 and 2024/2025 norovirus seasons. In all, 30 norovirus genotypes were identified among human norovirus cases; 96% (*n* = 19,731) of cases were GII, 4% (*n* = 845) were GI and <1% (*n* = 12) were GIX. GII.4 was the most frequently identified genotype, accounting for 54% of cases (*n* = 11,033). Of those with a determined variant type (*n* = 6205), 91% were GII.4 Sydney (*n* = 5668) (Table S3). Other GII.4 variants were found among cases, each accounting for ≤4% of cases, including Den Haag, Osaka and New Orleans during 2009/2010 to 2015/2016, Hong Kong during 2015/2016 to 2018/2019, and San Francisco, Wichita, and Allegany during 2019/2020 to 2024/2025 (Table S3). Other commonly detected genotypes included GII.3 (14%; *n* = 2928), GII.2 (9%; *n* = 1861), GII.17 (7%; *n* = 1444), and GII.6 (5%; *n* = 1115) (Figure 1). Other GII genotypes together comprised 7% (*n* = 1362) of cases (Figure S1). GI.3 was the most common GI genotype (46%; *n* = 390/845 of all GI cases) and in 2% (*n* = 390/20,588) of all norovirus cases (Figure S1).

**Figure 2:**
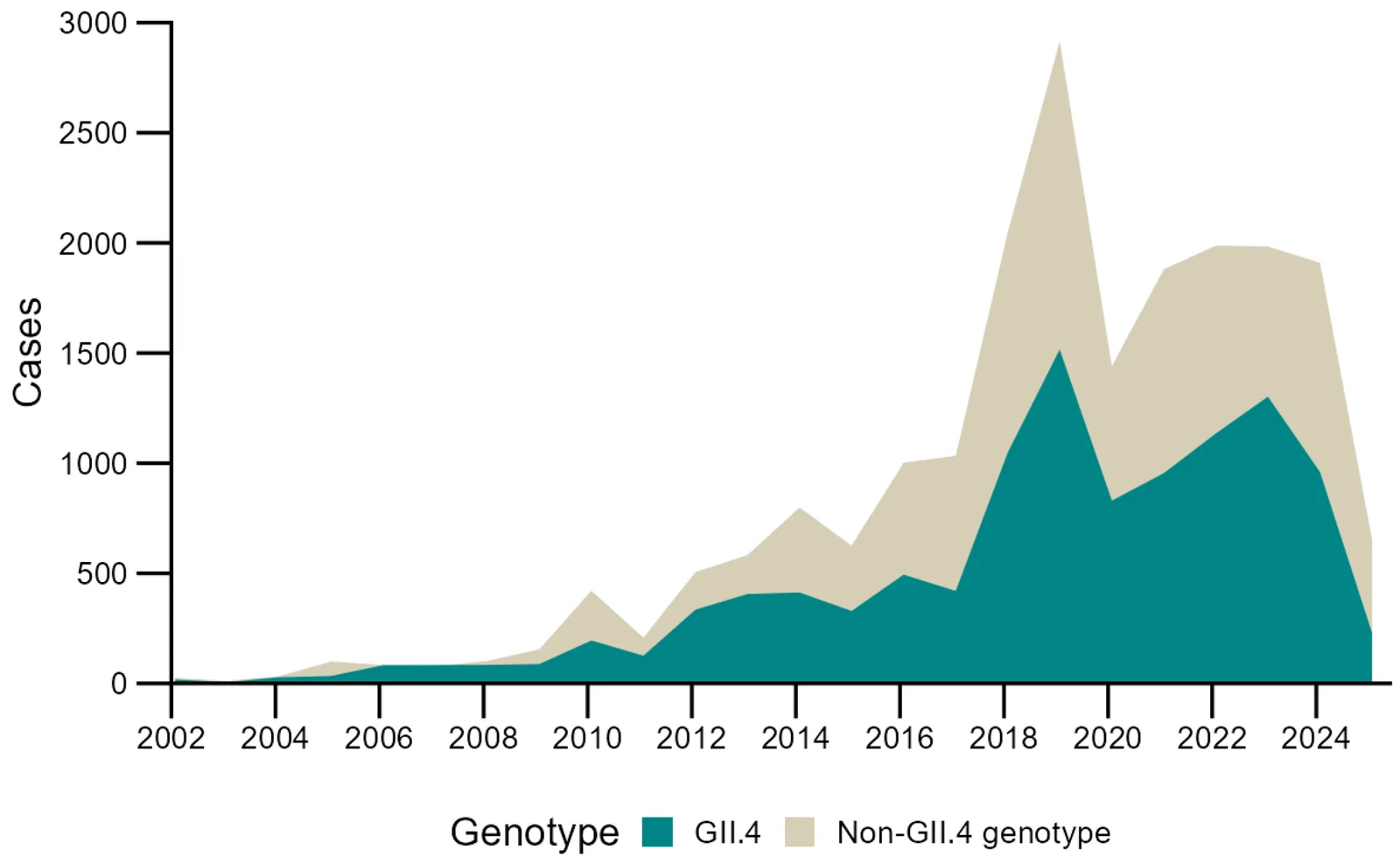
Case counts of GII.4 and non-GII.4 genotypes by norovirus season (*n* =20,588), 2002/2003–2024/2025.

Approximately 98% (*n* = 20,154) of norovirus cases had specimens collected from the 2008/2009 to the 2024/2025 norovirus seasons, with >150 cases represented in each season (median: 1002; range: 155–2916) (Figure 2). In contrast, substantially fewer cases were available for seasons before 2008/2009 (median, 79; range, 12–102); therefore, temporal analyses focused on the period from 2008/2009 onward (Figure 2).

**Figure 3:**
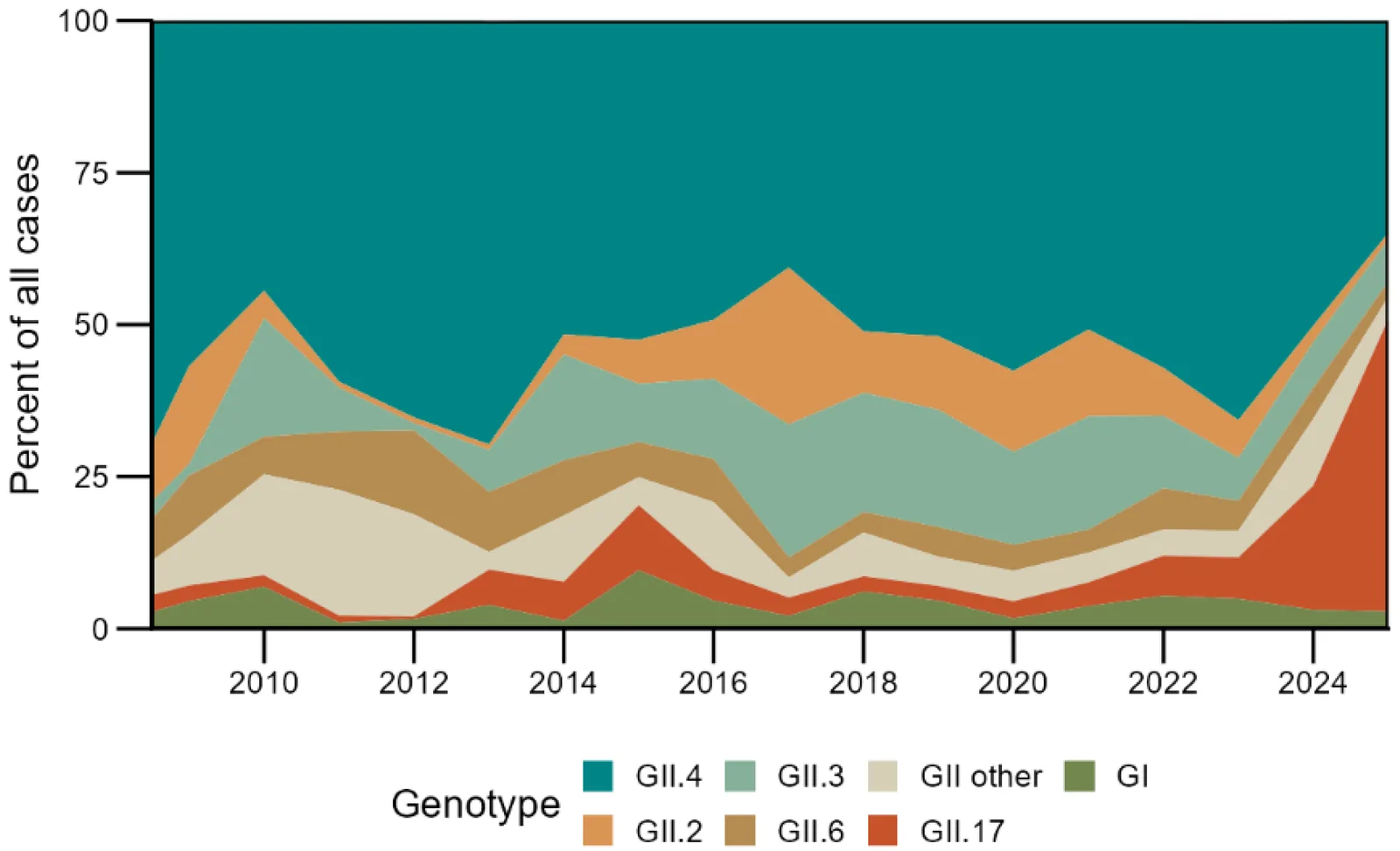
Prevalence of genotypes among norovirus cases (*n* = 20,154), by norovirus season 2008/2009–2024/2025. Data from 2002/2003–2007/2008 were excluded due of small sample sizes (<150 cases) making prevalence estimates less reliable.

GII.4 was the predominant genotype during most seasons but varied substantially in prevalence over time (Figure 3). It accounted for 57% of cases in 2008/2009 and exceeded 60% during several subsequent seasons, including 2010/2011 through 2012/2013. Periodic increases in other GII genotypes were also observed. GII.6 reached 14% in 2011/2012 before subsequently declining, while GII.3 increased to 17% in 2013/2014 and remained at ≥10% for the following eight seasons. GII.17 increased to 11% in 2014/2015, and GII.2 subsequently rose sharply, peaking at 26% (*n* = 267/1034) in 2016/2017. During the same season, GII.4 declined to 41% (*n* = 420/1034). GII.2 subsequently declined but remained at ≥10% for the next four seasons. GII.4 again became increasingly predominant in later seasons, reaching 66% (*n* = 1302/1984) in 2022/2023. This pattern shifted markedly in 2024/2025, when GII.4 declined to 35% (*n* = 229/653) and GII.17 increased to 48% (*n* = 314/653), making GII.17 the most frequently detected genotype in that season (Figure 3). GI viruses generally accounted for ≤5% of cases annually, with intermittent increases in 2009/2010, 2014/2015, and 2017/2018, largely attributable to GI.3 (Figure S2).

### 3.2. Distribution of Norovirus Genotypes by Age Category

An age category was available for 71% (*n* = 14,570/20,588) of cases derived from 58 publications, of which 97% (*n* = 14,109/14,570) were children (Table 1). The number of detected genotypes declined by age, with 28 in young children, 23 in children and adolescents, 14 in young adults, 13 in all adults and 13 in older adults (Table 1). In all, 20 genotypes were detected among adults (GI.1–6, GII.1–4, GII.6–10, GII.12–14, GII.17, and GII.21) and 28 among children (GI.1–9, GII.1–10, GII.12–14, GII.16–17, GII.20–21, GII.27 and GIX). GII viruses were found in 96% (*n* = 13,525/14,109) of cases among children and 92% (*n* = 424/461) of cases among adults (*p* < 0.0001). GI viruses were found among 4% (*n* = 572/14,109) of cases among children and 8% (*n* = 37/461) among adults (*p* < 0.0001). GII.4 prevalence was greater in adults (66%; *n* = 302/461) than in children (54%; *n* = 7569/14,109) (*p* < 0.0001), particularly among all adults (74%; *n* = 109/147) and older adults (80%; *n* = 98/123) categories (Table 1). All GII.4 variants detected in the dataset were found among children, whereas only GII.4 Sydney and GII.4 New Orleans were found among adults.

GII.2 prevalence did not significantly differ between child (7%; *n* = 1011/14,109) and adult 6% (*n* = 27/461) cases (*p* > 0.05), with 6% among young children (*n* = 542/8969), 9% among children & adolescents (*n* = 469/5140), and 12% among young adults (*n* = 22/191) but ≤2% for all adults (*n* = 3/147) and older adults (*n* = 2/123) (Table 1). Greater prevalence of GII.3 (*p* < 0.0001) and GII.17 (*p* < 0.001) was observed among children compared to adults. There was no significant difference in prevalence estimates for GII.6 or other GII viruses comparing child and adult cases (*p* > 0.05). GII.6 cases were 6% among young children (*n* = 566/8969) and 11% among young adults (*n* = 21/191), but ≤5% among children & adolescents and other adults (Table 1). Cases of other GII viruses were similarly dispersed among age groups (Table 1).

Adult cases had specimen collection dates almost entirely between the 2009/2010 and 2014/2015 norovirus seasons (Figure S3). During the 2009/2010 norovirus season, the overall peak in GII.3 was primarily driven by cases among children (Figure S3). Subsequently, the observed peak of GII.4 prevalence during the 2010/2011 to 2012/2013 seasons was more pronounced among adults than children, as was GI norovirus prevalence. Temporal trends in subsequent years, including the sharp rise in GII.17 viruses from the 2023/2024 and 2024/2025 norovirus seasons, were nearly entirely driven by cases among children (Figure S3).

### 3.3. Distribution of Norovirus Genotypes by Global Region

Case counts varied by region, with 68% in Asia/Oceania (*n* = 13,963/20,588), 12% (*n* = 2426) in North America, 9% in Central and South America (*n* = 1809), 10% in Europe (*n* = 2047), and 2% in Africa (*n* = 343) (Table 2). Genotype counts by region were highest in Asia/Oceania (*n* = 28) and Central and South America (*n* = 27), lowest in North America (*n* = 21) and Europe (*n* = 21), and, relative to small sample size (*n* = 343), moderately high in Africa (*n* = 23) (Table 2). In all, 96% (*n* = 19,743) of cases were GII and 4% (*n* = 845) were GI. GI viruses had the highest prevalence in Africa (9%; *n* = 32/343) and in Central and South America (9%; *n* = 168/1809) (Table 2, Figure S4). GII.4 predominated in all regions, ranging from 46% (*n* = 950/2047) in Europe and 49% Africa (*n* = 169/343) to 57% in North America (*n* = 1376/2426) (Table 2, Figure S4). GII.2 was most prevalent in Asia/Oceania (11%; *n* = 1529/13,963) and Europe (8%; *n* = 157/2047) (Table 2), but with the 2016/2017 peak activity primarily seen in North America and Asia/Oceania (Figure S4). GII.3 prevalence reached ≥5% in all regions except Central and South America (2%; *n* = 36/1809), with the highest prevalence in Europe (19%; *n* = 378/2047) and Asia/Oceania (16%; *n* = 2220/13,963) (Table 2), and with the 2009/2010 peak in activity primarily seen in Asia/Oceania (Figure S4). GII.6 prevalence was similar across regions. GII.17 reached ≥5% prevalence in all regions but was highest in Central and South America (12%; *n* = 217/1809) (Table 2). Peak GII.17 activity was seen across regions in 2023/2024 to 2024/2025 (Figure S4). Other GII viruses had the highest prevalence in Africa (17%; *n* = 58/343) but were also high in North America (12%; *n* = 283/2426) and Central and South America (12%; *n* = 221/1809) (Table 2; Figure S4). GII.4 was not found among adults in Africa, where 46% (*n* = 6/13) of cases among adults were GII.3. In Asia/Oceania and North America, GII.4 prevalence increased with age, reaching 86% (*n*= 72/84) and 84% (*n*= 26/31) of cases among older adults, respectively.

## 4. Discussion

A major challenge in characterizing global norovirus epidemiology is the lack of comprehensive surveillance data needed to monitor emerging strains, identify high-risk populations, and inform prevention strategies. This gap is particularly pronounced among adults, for whom available data were largely derived from outbreak surveillance. To address this limitation, we pooled data from 72 studies published over more than two decades that reported sporadic norovirus cases among children and adults across multiple geographic regions. GII.4 was the predominant genotype overall and across most age and geographic strata, accounting for 54% of all cases, consistent with previous global studies incorporating both outbreak-associated and sporadic cases [9,10,11,12]. We also observed an age-related increase in GII.4 prevalence, with the highest prevalence among older adults. This finding is consistent with previous outbreak surveillance showing a strong predominance of GII.4 in long-term care and other settings serving older populations [13].

Other frequently detected genotypes included GII.3, GII.2, GII.17, and GII.6, and their prevalence varied over time. Several temporal patterns observed in this analysis were consistent with previously reported genotype shifts. For instance, we found increased activity of GII.2 coinciding with the surges observed in Europe and Asia during the 2016/2017 norovirus season [10,19]. Periodic shifts in GII.3 activity [20,21] and GII.17 predominance in 2014/2015 and 2015/2016 as reported previously in China and Japan [22,23] were found, as well as a sharp increase in GII.17 activity during 2023/2024 and 2024/2025 norovirus seasons, when GII.17 predominated across many global regions, particularly in North America and Europe [16,24,25,26]. Consistent with our findings, these increases in non-GII.4 activity are typically short-lived (1–2 years), geographically limited, and followed by the re-establishment of GII.4 predominance [27]. Although published data were not available at the time of this literature review, publicly available surveillance data showed declining GII.17 activity in the 2025/2026 season. Declines were observed in outbreak and sporadic cases among adults and children captured in CaliciNet [28] in the US and from UKHSA [29] in the UK, as well as in global surveillance of sporadic cases among children <5 years of age in North America, Europe and Asia/Oceania as captured in NoroSurv [30]. However, IASR [31] data capturing outbreak and sporadic cases from adults and children in Japan show GII.17 remains predominant at the time of this review. However, delays in reporting across surveillance networks limit real-time monitoring of norovirus genetic trends. Furthermore, data largely reflect outbreak data or exclusively reflect sporadic norovirus infections in children <5 years of age and are limited in scope, with no demographic information or age stratification.

We found 30 genotypes (9 GI, 20 GII and 1 GIX) including all known GI and all but 3 (GII.23, GII.25, and GII.26) GII genotypes known to infect humans. Genotype diversity was greater among children than in adults and in regions with predominantly low- and middle-income countries (LMICs) (Central and South America and Africa) and Asia/Oceania, which included countries with a wide range of income levels [32]. GI.7–9, GII.5, GII.16, GII.20, GII.27 and GIX were found among children, but not among adults. GI.8–9 and GII.16, GII.20 and GII.27 were not found in higher income regions (Europe and North America). Genotype diversity among adults was consistent with Burke et al. (2019), where 24 genotypes were identified with a similar distribution and predominance of GII.4 in outbreaks primarily, but not exclusively, involving adults in healthcare settings [13]. GI norovirus prevalence was low overall (4%), with higher prevalence in Africa and Central and South America and among adults, with GI.3 predominating. Higher norovirus genotype diversity in low-income settings may reflect increased exposure and factors affecting immune development, including limited access to healthcare and sanitation, malnutrition and co-infections with other enteric pathogens [33,34]. However, interpretation of regional patterns is particularly challenging because many studies from low- and middle-income countries enrolled predominantly or exclusively children. Genotype diversity and prevalence of non-GII.4 genotypes (such as GII.2, GII.3 and GII.6) are reported to be higher among sporadic cases in children [9,12,35] than in outbreaks among adults [13], complicating regional comparisons. In this review, GII.3 prevalence was higher in sporadic cases among children than adults, but GII.2, GII.6 and other GII genotypes had similar prevalence in adults and children. We also found the overall prevalence of GII.17 was higher in children than adults, but since no published data were available from sporadic adult cases during the sharp rise in GII.17 activity observed in 2023/2024 and 2024/2025, this trend was likely due to data availability rather than a true difference between age groups. Indeed, the increase in GII.17 activity during the 2014/2015 norovirus season was seen in both adults and children.

Our review has several limitations. Cases were unevenly distributed across geographic regions and age groups, with pediatric populations overrepresented in the published literature and adult sporadic cases comparatively underrepresented. Adult data were especially limited in Central and South America, where studies included only children. While genotype diversity was greater among children, we cannot rule out the possibility that more genotypes would be detected among adults if greater sample sizes were available. Sample size limitations also precluded regional separation of subregions of Asia and Oceania, despite variation in country income levels. Although studies were screened for duplicate reporting, we cannot exclude the potential that some of the global surveillance data reported in NoroSurv [9,12] was also reported in country-specific publications. Case-level demographic data and precise sample collection dates were also unavailable, requiring reliance on aggregate genotype prevalence data across heterogeneous age ranges and study periods.

Approximately 93% of norovirus cases included in this review were reported from 2011/2012 onward and as such, nearly all GII.4 cases with an available variant type were GII.4 Sydney, precluding reliable comparison of GII.4 variant prevalence across age groups and geography. Furthermore, polymerase circulation could not be assessed, as few studies performed dual-typing of the polymerase and capsid. Future studies and surveillance networks should characterize GII.4 variants, as well as viral polymerases, as the emergence of novel variants and recombinant strains with new polymerases have been associated with increased norovirus activity and global spread [5,10,15]. An update of norovirus classification and nomenclature published in 2019 [8] reassigned some GII.22 viruses to GII.25 and GII.15 to genogroup GIX.1. As we limited our data extraction to GI and GII genogroups, only GIX viruses (reported in <1% of cases) previously reported as GII.15 were included. GII.25 presence in studies prior to 2019 could not be determined without norovirus sequences. As this review spanned decades of evolving molecular detection and genotyping methodologies, we cannot exclude the potential for genotype misclassification compared to current classification standards [8].

This study provides a comprehensive analysis of the global prevalence and diversity of norovirus genotypes in sporadic cases over two decades, confirming the global predominance of GII.4 and its higher prevalence among sporadic cases in adults compared to children. Other non-GII.4 GII genotypes (GII.3, GII.2, GII.17 and GII.6) and GI genotypes were consistently detected, with periods of increased activity and age-specific trends. Genotype diversity was greatest among children and in lower-income regions and Asia/Oceania.

These findings have important implications for norovirus vaccine development. The degree to which immunity following infection or vaccination protects against antigenically distinct noroviruses remains incompletely understood. Available evidence suggests that protection is generally greatest against antigenically related viruses, while the extent and durability of heterotypic protection across genotypes and genogroups remain uncertain [5,7,33]. Protection against GII.4 is further complicated by ongoing antigenic evolution and the periodic emergence of genetically and antigenically distinct variants [15]. Although GII.4 is included in several current vaccine candidates [5,36], the substantial circulation of non-GII.4 genotypes observed in this analysis suggests that the breadth of vaccine-induced protection will be an important determinant of population-level impact. This may be particularly relevant in populations and settings characterized by greater genotype diversity. Further research and clinical trials are needed to understand the breadth, duration and mechanisms of immunity following natural infection and vaccination. Our study highlights the need for continued expansion and harmonization of global genotype surveillance for detecting emerging strains, monitoring changes in genotype distribution, and informing future vaccine composition and public health strategies.

## Figures and Tables

**Table 1:** Norovirus cases and genotype distribution by age * (*n* = 14,570) across the 2002/2003 to 2024/2025 seasons.

	Young Children (≤5 years)	Children & Adolescents (6–15 years)	Young Adults (≥16 to 64 years)	All Adults (≥16 years)	Older Adults (≥65 years)
Number of cases	8969	5140	191	147	123
Number of genotypes	28	23	14	13	13
Genotype					
GII.4	52.4	55.8	49.7	74.1	79.7
GII.2	6.0	9.1	11.5	2.0	1.6
GII.3	12.7	18.9	6.3	4.1	3.3
GII.6	6.3	3.8	11.0	1.4	4.1
GII.17	9.5	3.2	1.6	3.4	3.3
GII other **	7.8	7.4	8.4	6.8	5.7
GI	5.4	1.8	11.5	8.2	2.4

* Age category definitions are indicated in Supplemental Table S2; Young children: ≤5 years, Children & adolescents: 6–15 years, Young adults: ≥16 to 64 years, All adults: ≥16 years, Older adults: ≥65 years, with some exceptions as indicated in Table S2. ** Category also includes GIX viruses (*n* = 12).

**Table 2:** Norovirus cases and genotype distribution by geographic region (*n* = 20,588) across the 2002/2003 to 2024/2025 seasons.

	North America	Central and South America	Europe	Africa	Asia/Oceania
Number of cases	2426	1809	2047	343	13,963
Number of genotypes	21	27	21	23	28
Genotype					
GII.4	56.7	52.7	46.4	49.3	54.3
GII.2	4.6	2.7	7.7	4.4	11.0
GII.3	11.2	2.0	18.5	6.4	15.9
GII.6	7.6	9.1	6.4	6.7	4.4
GII.17	5.0	12.0	9.1	7.0	6.4
GII other *	11.7	12.2	7.4	16.9	4.6
GI	3.2	9.3	4.5	9.3	3.4

* Category also includes GIX viruses (*n* = 12).

## Data Availability

Not applicable. No new data were created or analyzed in this study. All data are publicly available from the reference list.

## Supplementary Materials

The following supporting information can be downloaded at: https://www.mdpi.com/article/10.3390/v18101083/s1. References for the publications included in the literature review are found below [9,12,37,38,39,40,41,42,43,44,45,46,47,48,49,50,51,52,53,54,55,56,57,58,59,60,61,62,63,64,65,66,67,68,69,70,71,72,73,74,75,76,77,78,79,80,81,82,83,84,85,86,87,88,89,90,91,92,93,94,95,96,97,98,99,100,101,102,103,104,105,106].

## Abbreviations

The following abbreviations are used in this manuscript:

AGE	acute gastroenteritis
GI	genogroup 1 noroviruses
GII	genogroup 2 noroviruses
VP1	Viral protein 1
EMBASE	Excerpta Medica database
US	United States
UK	United Kingdom
UKHSA	United Kingdom Health Security Agency
IASR	Infectious Agents Surveillance Report
LMIC	low- and middle-income countries
