# Supplementary material for: Global Diversity of Norovirus Genotypes from 2002 to 2025: A Review of Temporal, Age-Specific and Regional Trends
Source: Viruses. 2026 Oct 1;18(10):1083. doi: 10.3390/v18101083 (PMC13709424; doi:10.3390/v18101083)
Supplement: Supplementary file 1 [file viruses-18-01083-s001.zip › viruses-4574586-supplementary.pdf]

Supplementary Material

Supplemental Text. Search strategies for literature review by PubMed and EMBASE (Ovid).

PubMed
#1 Norovirus[Mesh]
#2 ((norovirus*[Title/Abstract]) OR (Norwalk[Title/Abstract] AND Virus*[Title/Abstract])) OR (Small Round-Structured Virus*[Title/Abstract])
#3 #1 OR #2
#4 Serogroup[Mesh]
#5 serotype*[Title/Abstract] OR serogroup*[Title/Abstract] OR Serovar*[Title/Abstract] OR strain*[Title/Abstract]
#6 Genotype[Mesh]
#7 genotype*[Title/Abstract] OR genogroup*[Title/Abstract]
#8 #4 OR #5 OR #6 OR #7
#9 occurr*[Title/Abstract] OR circulat*[Title/Abstract]
#10 Disease Outbreaks[MeSH]
#11 Epidemiology[MeSH Subheading]
#12 outbreak*[Title/Abstract] OR surveillance[Title/Abstract]
#13 #9 OR #10 OR #11 OR #12
#14 #3 AND #8 AND #13 English, human

EMBASE
1 exp Norovirus/
2 norovirus*.ti,ab.
3 ((Norwalk or Small Round-Structured) adj2 Virus*).ti,ab.
4 or/1-3
5 serotype/
6 (serotype* or serogroup* or Serovar* or strain*).ti,ab.
7 exp genotype/
8 (genotype* or genogroup*).ti,ab.
9 or/5-8
10 (occurr* or circulat*).ti,ab.
11 epidemic/
12 Epidemiology/
13 (outbreak* or surveillance).ti,ab.
14 or/10-13
15 and/4,9,14
16 limit 15 to (human and english language and (article or article in press or "review"))
nb: for the May 2025 update preprints were also included in the Embase publication types.

Table S1. Included Studies

Author Year	Title	Region*	Country	Study Years	Reported Age	Age category*	Sample Size	PMID
Avellaneda 2025 [37]	High prevalence and genetic diversity of human norovirus among children under 5 years old with acute gastroenteritis at the Dr. Leonardo Guzmán Regional Hospital, Antofagasta, Chile, 2019	Central and South America	Chile	2019	<5 years	Young children	50	40573385
Badjo 2024 [38]	Genetic diversity of enteric viruses responsible of gastroenteritis in urban and rural Burkina Faso	Africa	Burkina Faso	2018-2021	0-85 years	All ages	20	38976836
Bruggink 2015 [39]	Norovirus genotype diversity in community-based sporadic gastroenteritis incidents: A five-year study	Asia/Oceania	Australia	2008-2012	0-15 years, 16-65 years, >65 years	Children & adolescents, Young adults and Older adults	91	25784155
Burke 2021 [40]	Norovirus and other viral causes of medically attended acute gastroenteritis across the age spectrum: results from the MAAGE Study in the United States	North America	US	2014-2016	<5 years, 5-17 years, 18-74 years, ≥75 years	Young children, Children & adolescents, Young adults and Older adults	218	34398953
Bykov 2024 [41]	Genetic diversity and phylogenetic relationship of human norovirus sequences derived from municipalities within the Sverdlovsk Region of Russia	Europe	Russia	2022-2023	2-7 years	Young children	196	39066164
Bykov 2025 [42]	Molecular characterization and epidemiology of human noroviruses in the Sverdlovsk Region, Russian Federation	Europe	Russia	2024	1-23 years	Children & adolescents	219	41012671
Campbell 2025 [43]	Incidence of norovirus-associated acute gastroenteritis across age groups in a Peruvian Andean community	Central and South America	Peru	2015-2018	0-99 years	All ages	99	39773432
Cannon 2021 [9]	Global trends in norovirus genotype distribution among children with acute gastroenteritis	Global	Global	2016-2020	<5 years	Young children	1,325	33900173

Cannon 2026 [12]	Global trends in norovirus genotype distribution among medically attended children with acute gastroenteritis, 2020-2025	Global	Global	2020-2025	<5 years	Young children	4108	42105550
Cao 2022 [44]	Molecular characteristics of norovirus in sporadic and outbreak cases of acute gastroenteritis and in sewage in Sichuan, China	Asia/Oceania	China	2015-2019	<5 years	Young children	163	36348433
Chan 2015 [45]	Virus genotype distribution and virus burden in children and adults hospitalized for norovirus gastroenteritis, 2012-2014, Hong Kong	Asia/Oceania	Hong Kong	2012-2014	≤5 years, 6-65 years, >65 years	Young children, All ages, and Older adults	317	26082165
Chen 2023 [46]	Molecular epidemiology analysis of symptomatic and asymptomatic norovirus infections in Chinese infants	Asia/Oceania	China	2021-2022	3- 4 months	Young children	14	37016444
Chen 2024 [47]	Epidemiological and genetic characteristics of norovirus in Hangzhou, China, in the post-epidemic era	Asia/Oceania	China	2021-2023	<18 years	Children & adolescents	81	38677156
Chen 2025 [48]	Molecular epidemiology of viral enteric in children in Hangzhou	Asia/Oceania	China	2019-2020	<14 years	Children & adolescents	105	41057835
Chilaúle 2022 [49]	Norovirus genetic diversity in children under five years old with acute diarrhea in Mozambique (2014-2015)	Global	Mozambique	2014-2015	<5 years	Young children	23	36146807
Currier 2015 [50]	Innate susceptibility to norovirus infections influenced by FUT2 genotype in a United States pediatric population	North America	US	2011-2012	<5 years	Young children	255	25744498
da Costa 2024 [51]	Epidemiological and molecular surveillance of norovirus in the Brazilian Amazon: description of recombinant genotypes and improvement of evolutionary analysis	Central and South America	Brazil	2018-2022	All ages	All ages	53	38656038
Eftekhari 2023 [52]	The predominance of recombinant Norovirus GII.4 Sydney[P16] strains in children less than 5	Asia/Oceania	Iran	2021-2022	<5 years	Young children	35	37459917

	years of age with acute gastroenteritis in Tehran, Iran, 2021–2022							
Ennuschat 2021 [53]	Norovirus epidemiology and genetic diversity in Leipzig, Germany during 2013-2017	Europe	Germany	2013-2017	<5 years	Young children	270	34696390
Epifanova 2022 [54]	An increase in prevalence of recombinant GII.3[P12] norovirus in sporadic acute diarrhea in children in Nizhny Novgorod, Russia, 2018–2021	Europe	Russia	2018-2021	NR	All ages	355	35680691
Fang 2021 [55]	Molecular epidemiology of norovirus infections in children with acute gastroenteritis in 2017–2019 in Tianjin, China	Asia/Oceania	China	2017-2019	≤12 years	Children & adolescents	785	34528724
Gao 2011 [56]	Clinical and molecular epidemiologic analyses of norovirus-associated sporadic gastroenteritis in adults from Beijing, China	Asia/Oceania	China	2007-2008	≥18 years	All adults	45	21503924
Gentile 2023 [57]	Role of noroviruses in sporadic acute gastroenteritis cases from children attending a large referral children’s hospital in Buenos Aires City, Argentina	Central and South America	Argentina	2017-2021	<5 years	Young children	51	36638391
Giri 2024 [58]	Hospital-based norovirus surveillance in children <5 years of age from 2017 to 2019 in India	Asia/Oceania	India	2017-2019	<5 years	Young children	219	38235830
Gomes 2025 [59]	Norovirus in pediatric gastroenteritis: a study in Argentine hospitals before and after the introduction of universal rotavirus vaccination	Central and South America	Argentina	2011-2012, 2019-2020	<5 years	Young children	29	41295453
Gyrtdal 2015 [60]	Incidence of medically-attended norovirus-associated acute gastroenteritis in four Veteran's Affairs medical center populations in the United States, 2011-2012	North America	US	2011-2012	20-96 years	All adults	50	
Haddadin 2021 [61]	Characteristics of GII.4 norovirus versus other genotypes in sporadic pediatric infections in Davidson County, Tennessee, USA	North America	US	2012-2015	<18 years	Children & adolescents	460	32667045

Hassan 2019 [62]	Viral etiology of acute gastroenteritis in <2-year-old US children in the post-rotavirus vaccine era	North America	US	2012	<2 years	Young children	58	30184153
Honjo 2022 [63]	Genotypes and transmission routes of noroviruses causing sporadic acute gastroenteritis among adults and children, Japan, 2015-2019	Asia/Oceania	Japan	2015-2019	<16 years	Children & adolescents	122	35952938
John 2021 [64]	High proportion of norovirus infection and predominance of GII.3 [P12] genotype among the children younger than 5 in Sabah, Malaysian Borneo	Asia/Oceania	Malaysia	2018-2019	<5 years	Young children	38	34509928
Kabue 2023 [65]	Norovirus-associated gastroenteritis Vesikari score and pre-existing salivary IgA in young children from rural South Africa	Africa	South Africa	2019-2021	<5 years	Young children	21	38005863
Kambhampati 2019 [66]	Active surveillance for norovirus in a US Veterans Affairs patient population, Houston, Texas, 2015-2016	North America	US	2015-2016	≥18 years	All adults	14	30949545
Kébé 2022 [67]	Prevalence and genetic characterization of noroviruses in children with acute gastroenteritis in Senegal, 2007–2010	Africa	Senegal	2007-2010	<5 years	Young children	58	34854097
Kébé 2026 [68]	A sixteen-month monitoring of human enteric viruses associated with acute gastroenteritis in Senegal	Africa	Senegal	2020-2021	All ages	All ages	21	41680924
Khamrin 2022 [69]	Changing predominance of norovirus recombinant strains GII.2[P16] to GII.4[P16] and GII.4[P31] in Thailand, 2017 to 2018	Asia/Oceania	Thailand	2017-2018	<5 years	Young children	131	35546545
Khamrin 2024 [70]	Genetic recombination and genotype diversity of norovirus GI in children with acute gastroenteritis in Thailand, 2015-2021	Asia/Oceania	Thailand	2015-2021	<15 years	Children & adolescents	392	38237357

Kittigul 2025 [71]	Emergence of norovirus GII.17[P16] in adult patients with acute gastroenteritis in Thailand during 2021−2023	Asia/Oceania	Thailand	2021-2023	12 to 84	All ages	28	41284670
Kõivumägi 2022 [72]	Norovirus strains among children aged 0–18 years hospitalized with acute gastroenteritis in Estonia 2015–2016	Europe	Estonia	2015-2016	<18 years	Children & adolescents	189	34854093
Lanata 2025 [73]	Epidemiology of norovirus disease in the first 2 years of life: a prospective multisite cohort study in Lima, Peru	Central and South America	Peru	2019-2020	1-2 years	Young children	156	39579923
Lee 2025 [74]	Trends in norovirus genotypes in South Korea, 2019–2024: insights from nationwide dual typing surveillance	Asia/Oceania	South Korea	2019-2024	All ages	All ages	2,622	41472243
Li 2023 [75]	Epidemiology and genetic diversity of norovirus GII genogroups among children in Hubei, China, 2017–2019	Asia/Oceania	China	2017-2019	≤5 years	Young children	131	37030436
Lo 2024 [76]	Comprehensive full genome analysis of norovirus strains from eastern India, 2017–2021	Asia/Oceania	India	2017–2021	All ages	All ages	40	38238807
Lu 2023 [77]	Changing predominance of norovirus strains in children with acute gastroenteritis in Shanghai, 2018–2021	Asia/Oceania	China	2018-2021	≤16 years	Children & adolescents	180	37619918
Lu 2025 [78]	Resurgence and predominance of GII.17[P17] norovirus in children with sporadic acute gastroenteritis in Shanghai, China	Asia/Oceania	China	2022-2024	<16 years	Children & adolescents	272	41328700
Luchs 2025 [79]	Concurrent circulation of viral agents in pediatric patients presenting with respiratory illness and diarrheal symptoms in Metropolitan Region of Sao Paulo, Brazil, 2021	Central and South America	Brazil	2021	≤3 years	Young children	13	40284939
Ly 2026 [80]	Clinical, molecular and evolutionary characterization of noroviruses in hospitalized children with acute gastroenteritis in Vietnam, 2016 to 2021	Asia/Oceania	Vietnam	2016-2021	<5 years	Young children	406	41863254

Ma 2024 [81]	Prevalence and genotype distribution of norovirus in Ningxia Hui Autonomous Region, China, from 2011 to 2022	Asia/Oceania	China	2011-2022	All ages	All ages	1,786	39334155
Ofosu-Appiah 2025 [82]	Clinical impact and genetic analysis of enteric viruses associated with acute gastroenteritis in Greater Accra, Ghana: a comprehensive study of five viruses	Africa	Ghana	2019	0-75 years	All ages	5	39935201
Ortiz-Quintero 2024 [83]	Molecular characterization of enteric viruses causing acute gastroenteritis among children under 5 years old in Distrito Central, Honduras	Central and South America	Honduras	2010-2011	<5 years	Young children	11	38981462
Ozawa 2007 [84]	Norovirus infections in symptomatic and asymptomatic food handlers in Japan	Asia/Oceania	Japan	2005-2006	Not reported	All ages	35	17928420
Payne 2013 [85]	Norovirus and medically attended gastroenteritis in U.S. children	North America	US	2010	<5 years	Young children	100	23514289
Pham 2023 [86]	Genetic diversity and declining norovirus prevalence in infants and children during Japan's COVID-19 pandemic: a three-year molecular surveillance	Asia/Oceania	Japan	2018-2021	<16 years	Children & adolescents	355	37584776
Phengma 2022 [87]	Molecular epidemiology and characterization of norovirus and sapovirus in pediatric patients with acute diarrhea in Thailand, 2019-2020	Asia/Oceania	Thailand	2019-2020	<5 years	Young children	127	35994998
Pimenta 2025 [88]	Epidemiological profiles of norovirus genotypes in children with and without acute gastroenteritis from the northwestern Amazon region	Central and South America	Brazil	2016-2017	<5 years	Young children	69	40730322
Pitkänen 2022a [89]	A major decrease in viral acute gastroenteritis in hospitalized Finnish children as rotavirus returns as the most detected pathogen	Europe	Finland	2017-2018	<16 years	Children & adolescents	48	34775115

Pitkänen 2022b [90]	Sapovirus, norovirus and rotavirus detections in stool samples of hospitalized Finnish children with and without acute gastroenteritis	Europe	Finland	2009-2011	<16 years	Children & adolescents	106	35185141
Reyes 2022 [91]	Secretor status strongly influences the incidence of symptomatic norovirus infection in a genotype-dependent manner in a Nicaraguan birth cohort	Central and South America	Nicaragua	2017-2020	<3 years	Young children	114	34129046
Sai 2013 [92]	Clinical and molecular epidemiology of norovirus infection in adults with acute gastroenteritis in Ji'nan, China	Asia/Oceania	China	2010-2011	>16 years	All adults	39	23754743
Sakon 2015 [93]	Impact of genotype-specific herd immunity on the circulatory dynamism of norovirus: a 10-year longitudinal study of viral acute gastroenteritis	Asia/Oceania	Japan	2002-2012	0-14 years	Children & adolescents	631	25210139
Sharif 2023 [94]	High prevalence of norovirus GII.4 Sydney among children with acute gastroenteritis in Bangladesh, 2018–2021	Asia/Oceania	Bangladesh	2018-2021	<5 years	Young children	27	37178475
Supadej 2019 [95]	Distribution of norovirus and sapovirus genotypes with emergence of NoV GII.P16/GII.2 recombinant strains in Chiang Mai, Thailand	Asia/Oceania	Thailand	2015-2017	<16 years	Children & adolescents	173	29995327
Tang 2013 [96]	Epidemiological and molecular analysis of human norovirus infections in Taiwan during 2011 and 2012	Asia/Oceania	Taiwan	2011-2012	All ages	All ages	7	23875971
Tegegne 2024 [97]	Genetic diversity and distribution of noroviruses among all age groups of patients with diarrhea in Amhara National Regional State, Ethiopia	Africa	Ethiopia	2021	<5, 4-17, 18-64, >64 years	Young children, Children & adolescents, Young adults and Older adults	29	38771749
Umair 2025 [98]	Molecular epidemiology and genomic insights into norovirus and coinfections: a 2023 tertiary care hospital study on pediatric acute gastroenteritis in Pakistan	Asia/Oceania	Pakistan	2023	<5 years	Young children	7	40720842

Wang 2019 [99]	Norovirus activity and genotypes in sporadic acute diarrhea in children in Shanghai during 2014-2018	Asia/Oceania	China	2014-2018	≤16 years	Children & adolescents	348	31626043
Wang 2025 [100]	Genetic diversity of norovirus in Shenzhen based on continuous surveillance from 2016 to 2022	Asia/Oceania	China	2020-2022	All ages	All ages	172	40606629
Wu 2015 [101]	Prevalence and genetic diversity of noroviruses in adults with acute gastroenteritis in Huzhou, China, 2013-2014	Asia/Oceania	China	2013-2014	16-59, >60 years	Young adults, Older adults	68	25951970
Yan 2025 [102]	Molecular epidemiology and evolution of norovirus in Australia and New Zealand, 2018 to 2020	Asia/Oceania	Australia and New Zealand	2018-2020	Not reported	All ages	534	40100222
Yi 2016 [103]	Molecular epidemiology of norovirus in children and the elderly in Atlanta, Georgia, United States	North America	US	2012-2013	≤18 years, ≥65 years	Children & adolescents, Older adults	63	26600094
Yu 2026 [104]	The increased detection of recombinant norovirus GII.4 Sydney 2012[P16] strains in hospitalized pediatric patients with gastroenteritis in Shanghai, China	Asia/Oceania	China	2018-2021	<16 years	Children & adolescents	603	41606719
Zheng 2026 [105]	Increase of GII.17 norovirus infections during the 2024-2025 season in multiple cities, China	Asia/Oceania	China	2024-2025	≤6 years	Young children	412	41770883
Zhu 2025 [106]	A new wave of resurgence for GII.4 Sydney in Huzhou, particularly GII.4 Sydney[P16], between 2019 and 2023	Asia/Oceania	China	2019-2023	All ages	All ages	212	39972255

* Region and age categories were defined in this review. See Table S2 for age category definitions. The “All ages” category was defined as studies where age was not reported or the all ages (0-99+) were included without stratification; these data were excluded from the age specific analyses.

Table S2. Definitions for age categories

Age group	Age range (years)
Young children	≤5*
Children & adolescents	6–15**
Young adults	≥16-64***
All adults	≥16***
Older adults	≥65****

*One study included children 0-7 years
**One study included children 1-23 years
***Considered adults if >16 years or ≥17 years
****Considered older adults also if >60 years or >74 years

Table S3. Distribution of GII.4 variants among norovirus cases, from the 2002/2003 to the 2024/2025 norovirus seasons.

GII.4 variant	Number	Percent of total GII.4 (n = 11,033)	Percent of total with GII.4 variant assigned (n =6,205)
Unknown/not typed	4,828	43.8%	NA
Sydney	5,668	51.4%	91.3%
New Orleans	234	2.1%	3.8%
San Francisco	178	1.6%	2.9%
Wichita	60	0.5%	1.0%
Den Haag	36	0.3%	0.6%
Allegany	17	0.2%	0.3%
Hong Kong	11	0.1%	0.2%
Osaka	1	0.00%	0.01%

* NA; not applicable

Figure S1. Distribution of norovirus genotypes comprising <5% of all norovirus cases (n=20,588), from the 2002/2023 to the 2024/2025 norovirus seasons., GII.23, GII.25, GII.26

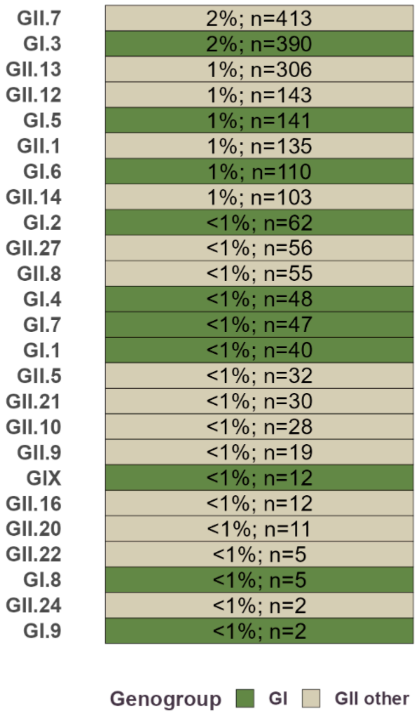

Figure S2. Distribution of other norovirus genotypes each representing <5% of overall cases (n=20,154), by norovirus season, 2008/2009 to 2024/2025.

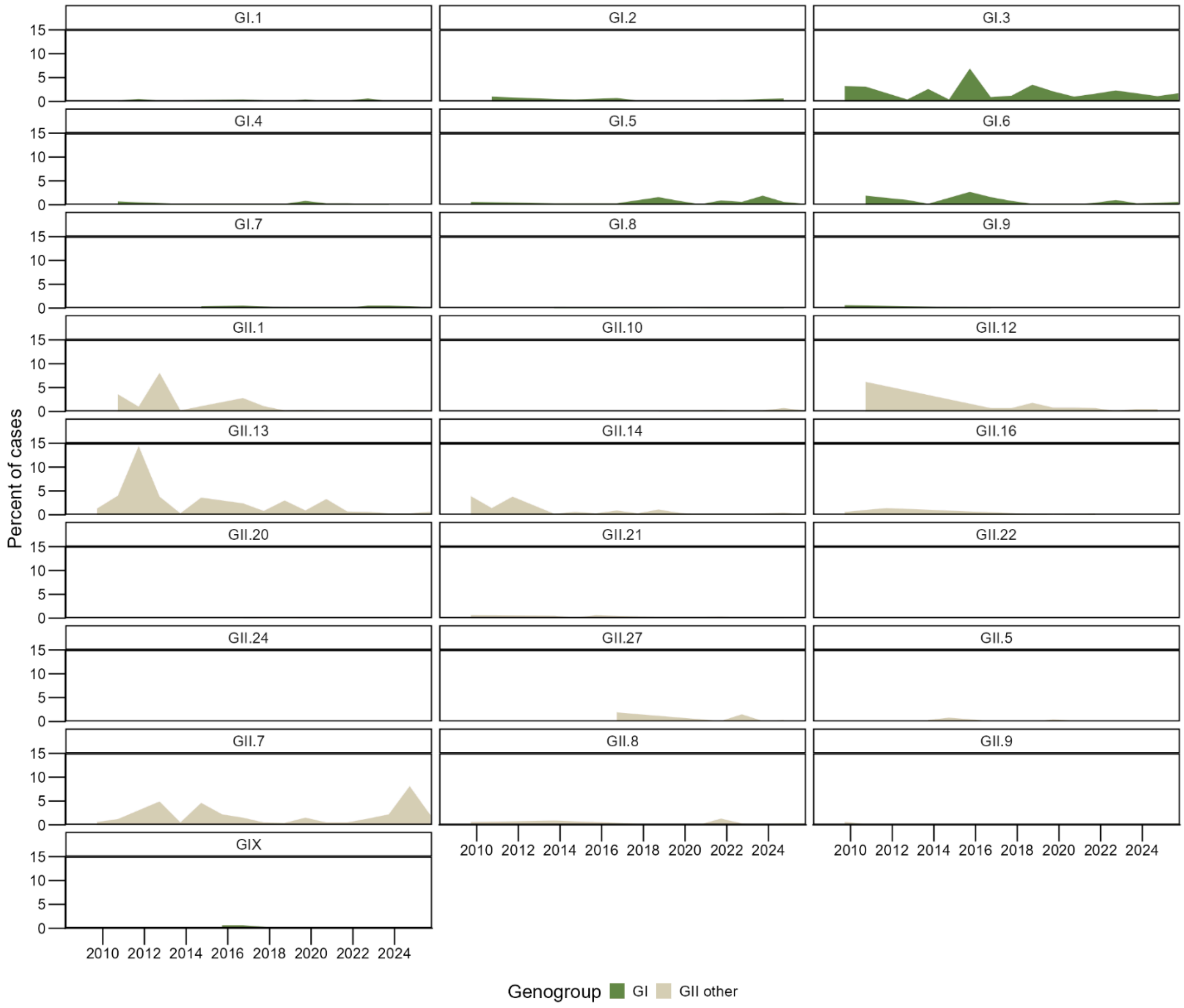

Figure S3. Norovirus cases and genotype distribution (n=20,154), by norovirus season 2008/2009–2024/2025 for A) Children and B) Adults.

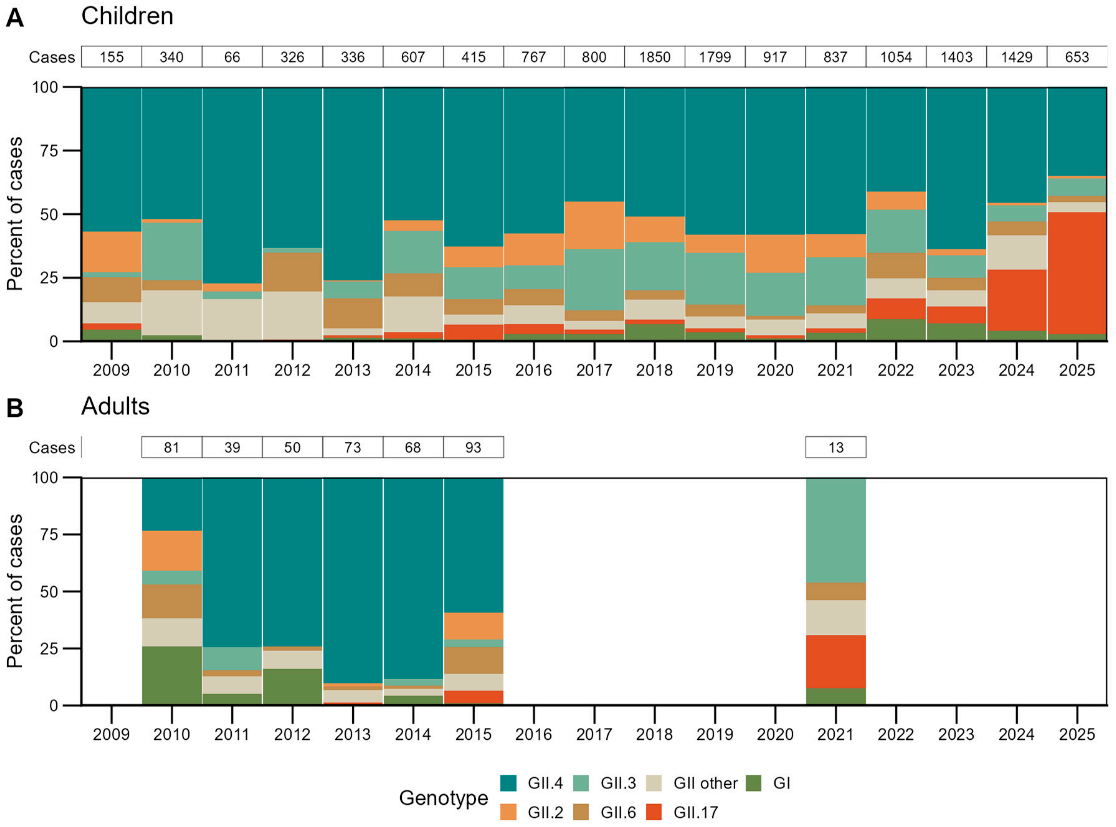

Figure S4. Norovirus cases and genotype distribution (n=20,154), by norovirus season 2008/2009–2024/2025 for A) North America, B) Central and South America, C) Europe, D) Africa, E) Asia/Oceania.

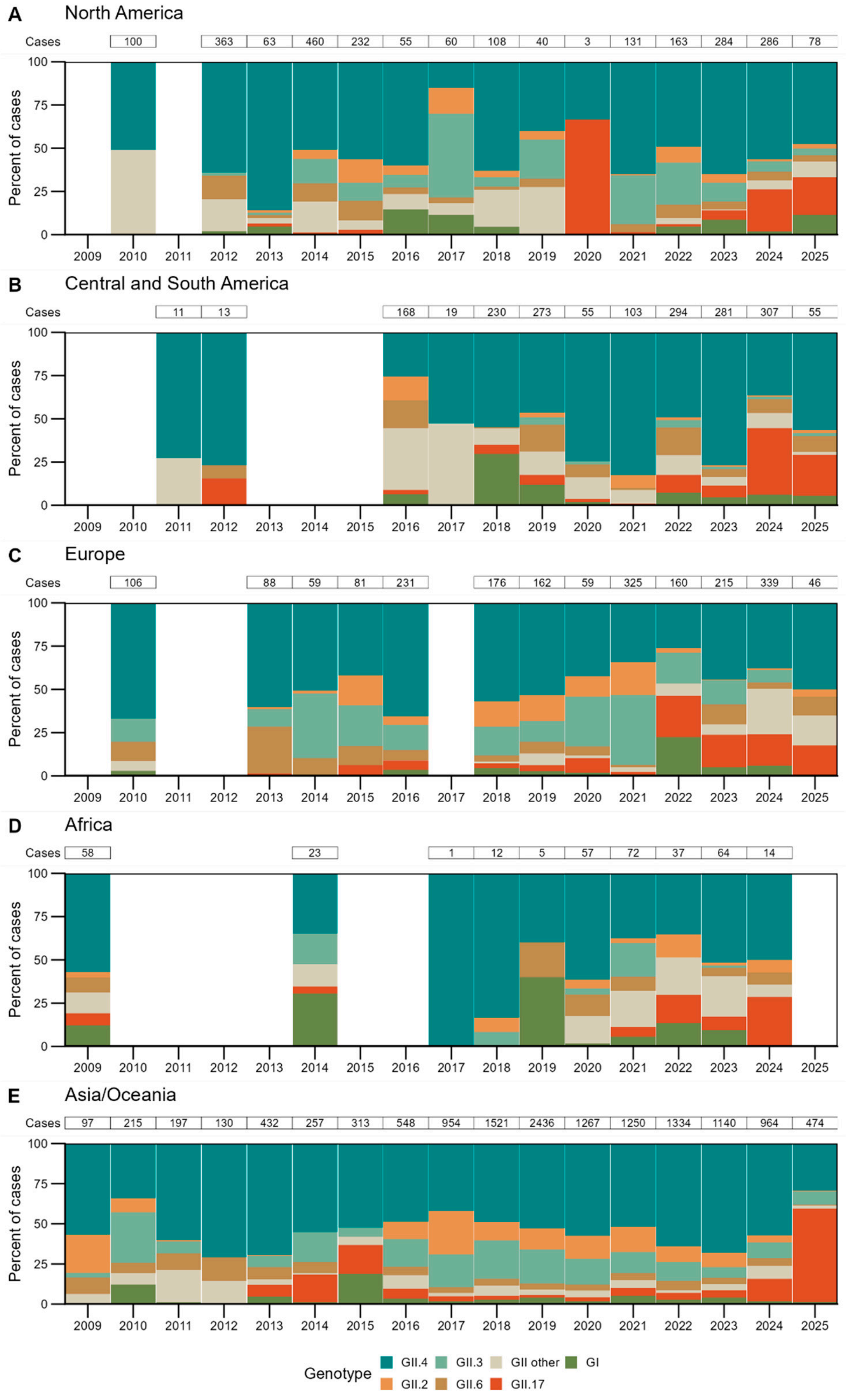

References

9. Cannon, J.L.; Bonifacio, J.; Bucardo, F.; Buesa, J.; Bruggink, L.; Chan, M.C.-W.; Fumian, T.M.; Giri, S.; Gonzalez, M.D.; Hewitt, J.; et al. Global Trends in Norovirus Genotype Distribution Among Children with Acute Gastroenteritis. *Emerg Infect Dis* **2021**, *27*, 1438–1445, doi:10.3201/eid2705.204756.

12. Cannon, J.L.; Bonifacio, J.; Fumian, T.M.; Pabbaraju, K.; Pietsch, C.; Gonzalez, M.D.; Hossain, M.E.; Selvarangan, R.; Buesa, J.; Pan, C.-Y.; et al. Global Trends in Norovirus Genotype Distribution Among Medically Attended Children with Acute Gastroenteritis, 2020-2025. *J Clin Virol* **2026**, *184*, 105949, doi:10.1016/j.jcv.2026.105949.

37. Avellaneda, A.M.; Campillay-Véliz, C.P.; Reyes, D.C.; Herrera, D.; Muñoz, C.A.; Vinjé, J.; Lay, M.K. High Prevalence and Genetic Diversity of Human Norovirus Among Children Under 5 Years Old with Acute Gastroenteritis at the Dr. Leonardo Guzmán Regional Hospital, Antofagasta, Chile, 2019. *Viruses* **2025**, *17*, 794, doi:10.3390/v17060794.

38. Badjo, A.O.R.; Niendorf, S.; Jacobsen, S.; Zongo, A.; Mas Marques, A.; Vietor, A.C.; Kabore, N.F.; Poda, A.; Some, S.A.; Ouattara, A.; et al. Genetic Diversity of Enteric Viruses Responsible of Gastroenteritis in Urban and Rural Burkina Faso. *PLoS Negl Trop Dis* **2024**, *18*, e0012228.

39. Bruggink, L.D.; Dunbar, N.L.; Marshall, J.A. Norovirus Genotype Diversity in Community-Based Sporadic Gastroenteritis Incidents: A Five-Year Study. *J Med Virol* **2015**, *87*, 961–969, doi:10.1002/jmv.24154.

40. Burke, R.M.; Mattison, C.P.; Marsh, Z.; Shioda, K.; Donald, J.; Salas, S.B.; Naleway, A.L.; Biggs, C.; Schmidt, M.A.; Hall, A.J. Norovirus and Other Viral Causes of Medically Attended Acute Gastroenteritis Across the Age Spectrum: Results from the Medically Attended Acute Gastroenteritis Study in the United States. *Clin Infect Dis* **2021**, *73*, e913–e920, doi:10.1093/cid/ciab033.

41. Bykov, R.; Itani, T.; Starikova, P.; Skryabina, S.; Kilyachina, A.; Koltunov, S.; Romanov, S.; Semenov, A. Genetic Diversity and Phylogenetic Relationship of Human Norovirus Sequences Derived from Municipalities within the Sverdlovsk Region of Russia. *Viruses* 2024, *16*.

42. Bykov, R.; Itani, T.; Pletenchuk, D.; Ohlopkova, O.; Moshkin, A.; Stepanyuk, M.; Semenov, A. Molecular Characterization and Epidemiology of Human Noroviruses in the Sverdlovsk Region, Russian Federation. *Viruses* **2025**, *17*, doi:10.3390/v17091243.

43. Campbell, W.R.; Neyra, J.; Calderwood, L.E.; Romero, C.; Soto, G.; Kambhampati, A.K.; Hall, A.J.; Ponce, D.; Galvan, P.; Tinoco, Y.O.; et al. Incidence of Norovirus-Associated Acute Gastroenteritis across Age Groups in a Peruvian Andean Community. *American Journal of Tropical Medicine and Hygiene* **2025**, *112(4)*, 921–927.

44. Cao, R.; Ma, X.; Pan, M. Molecular Characteristics of Norovirus in Sporadic and Outbreak Cases of Acute Gastroenteritis and in Sewage in Sichuan, China. *Virol J* **2022**, *19*, 180, doi:10.1186/s12985-022-01897-w.

45. Chan, M.C.W.; Leung, T.F.; Chung, T.W.S.; Kwok, A.K.; Nelson, E.A.S.; Lee, N.; Chan, P.K.S. Virus Genotype Distribution and Virus Burden in Children and Adults Hospitalized for Norovirus Gastroenteritis, 2012-2014, Hong Kong. *Sci Rep* **2015**, *5*, 11507, doi:10.1038/srep11507.

46. Chen, L.N.; Wang, S.J.; Wang, S.M.; Fu, X.L.; Zheng, W.J.; Hao, Z.Y.; Zhou, H.S.; Zhang, X.J.; Zhao, Y.L.; Qiu, C.; et al. Molecular Epidemiology Analysis of Symptomatic and Asymptomatic Norovirus Infections in Chinese Infants. *Virol J* **2023**, *20*, 60, doi:10.1186/s12985-023-02024-z.

47. Chen, D.; Shao, Q.; Ru, X.; Chen, S.; Cheng, D.; Ye, Q. Epidemiological and Genetic Characteristics of Norovirus in Hangzhou, China, in the Post-epidemic Era. *J Clin Virol* **2024**, *172*, 105679, doi:10.1016/j.jcv.2024.105679.

48. Chen, B.H.; Lai, Q.R.; Chen, Y.Y.; Zhou, H.Y.; Cai, D.T.; Shen, H.Q.; Li, W. Molecular Epidemiology of Viral Enteric in Children in Hangzhou. *BMC Infect Dis* **2025**, *25*, 1243, doi:10.1186/s12879-025-11678-9.

49. Chilaúle, J.J.; Munlela, B.; Mans, J.; Mabasa, V.V.; Marques, S.; Bauhofer, A.F.L.; Jane, G.; Anapakala, E.; Oliveira, F.; Cossa-Moiane, I.; et al. Norovirus Genetic Diversity in Children under Five Years Old with Acute Diarrhea in Mozambique (2014-2015). *Viruses* **2022**, *14*, 2001, doi:10.3390/v14092001.

50. Currier, R.L.; Payne, D.C.; Staat, M.A.; Selvarangan, R.; Shirley, S.H.; Halasa, N.; Boom, J.A.; Englund, J.A.; Szilagyi, P.G.; Harrison, C.J.; et al. Innate Susceptibility to Norovirus Infections Influenced by FUT2 Genotype in a United States Pediatric Population. *Clin Infect Dis* **2015**, *60*, 1631–1638, doi:10.1093/cid/civ165.
51. da Costa, J.N.; Siqueira, J.A.M.; Teixeira, D.M.; Lobo, P.S.; Guerra, S.F.S.; Souza, I.M.; Cardoso, B.T.M.; Farias, L.S.S.; Resque, H.R.; Gabbay, Y.B.; et al. Epidemiological and Molecular Surveillance of Norovirus in the Brazilian Amazon: Description of Recombinant Genotypes and Improvement of Evolutionary Analysis. *Revista do Instituto de Medicina Tropical de Sao Paulo* **2024**, *66(no pagination)*.
52. Eftekhari, M.; Kachooei, A.; Jalilvand, S.; Latifi, T.; Habib, Z.; Ataei-Pirkoohi, A.; Marashi, S.M.; Shoja, Z. The Predominance of Recombinant Norovirus GII.4 Sydney[P16] Strains in Children Less than 5 Years of Age with Acute Gastroenteritis in Tehran, Iran, 2021-2022. *Virus Res* **2023**, *334*, 199172, doi:10.1016/j.virusres.2023.199172.
53. Ennuschat, N.; Härtel, S.; Pietsch, C.; Liebert, U.G. Norovirus Epidemiology and Genetic Diversity in Leipzig, Germany during 2013-2017. *Viruses* **2021**, *13*, 1961, doi:10.3390/v13101961.
54. Epifanova, N.V.; Sashina, T.A.; Morozova, O.V.; Oparina, S.V.; Novikova, N.A. An Increase in Prevalence of Recombinant GII.3[P12] Norovirus in Sporadic Acute Diarrhea in Children in Nizhny Novgorod, Russia, 2018-2021. *Virus Genes.* **2022**, doi:https://dx.doi.org/10.1007/s11262-022-01919-3.
55. Fang, Y.; Dong, Z.; Liu, Y.; Wang, W.; Hou, M.; Wu, J.; Wang, L.; Zhao, Y. Molecular Epidemiology and Genetic Diversity of Norovirus Among Hospitalized Children with Acute Gastroenteritis in Tianjin, China, 2018-2020. *BMC Infect Dis* **2021**, *21*, 682, doi:10.1186/s12879-021-06375-2.
56. Gao, Y.; Jin, M.; Cong, X.; Duan, Z.; Li, H.Y.; Guo, X.; Zuo, Y.; Zhang, Y.; Zhang, Y.; Wei, L. Clinical and Molecular Epidemiologic Analyses of Norovirus-Associated Sporadic Gastroenteritis in Adults from Beijing, China. *J Med Virol* **2011**, *83*, 1078–1085, doi:10.1002/jmv.22077.
57. Gentile, Á.; Areso, M.S.; Degiuseppe, J.I.; Orqueda, A.; Turco, M.; Sabbaj, L.; Rodrigo, A.; Juárez, M.D.V.; Stupka, J.A. Role of Noroviruses in Sporadic Acute Gastroenteritis Cases from Children Attending a Large Referral Children's Hospital in Buenos Aires City, Argentina. *Pediatr Infect Dis J* **2023**, *42*, 94–98, doi:10.1097/inf.0000000000003775.
58. Giri, S.; Chhabra, P.; Kulkarni, R.; Reju, S.; Sabapathy, S.K.; Selvarajan, S.; Varghese, T.; Kalaivanan, M.; Dorairaj, P.; Kalrao, V.; et al. Hospital-Based Norovirus Surveillance in Children <5 Years of Age from 2017 to 2019 in India. *J Med Virol* **2024**, *96*, e29384, doi:10.1002/jmv.29384.
59. Gomes, K.A.; Rivero, K.A.; Barrios Mathieur, C.; Degiuseppe, J.I.; Cortes, P.R.; Gonzalez, P.A.; Zurschmitten, A.; Castro, M.P.; Parreno, V.; Mozgovoj, M.V.; et al. Norovirus in Pediatric Gastroenteritis: A Study in Argentine Hospitals Before and After the Introduction of Universal Rotavirus Vaccination. *Vaccines* **2025**, *13(11) (no pagination)*, doi:10.3390/vaccines13111080.
60. Grytdal, S.P.; Rimland, D.; Shirley, S.H.; Rodriguez-Barradas, M.C.; Goetz, M.B.; Brown, S.T.; Lucero-Obusan, C.; Holodniy, M.; Graber, C.; Parashar, U.; et al. Incidence of Medically-Attended Norovirus-Associated Acute Gastroenteritis in Four Veteran's Affairs Medical Center Populations in the United States, 2011-2012. *PLoS One* **2015**, *10*, e0126733, doi:10.1371/journal.pone.0126733.
61. Haddadin, Z.; Batarseh, E.; Hamdan, L.; Stewart, L.S.; Piya, B.; Rahman, H.; Spieker, A.J.; Chappell, J.; Wikswo, M.E.; Dunn, J.R.; et al. Characteristics of GII.4 Norovirus Versus Other Genotypes in Sporadic Pediatric Infections in Davidson County, Tennessee, USA. *Clin Infect Dis* **2021**, *73*, e1525–e1531, doi:10.1093/cid/ciaa1001.
62. Hassan, F.; Kanwar, N.; Harrison, C.J.; Halasa, N.B.; Chappell, J.D.; Englund, J.A.; Klein, E.J.; Weinberg, G.A.; Szilagyi, P.G.; Moffatt, M.E.; et al. Viral Etiology of Acute Gastroenteritis in <2-Year-Old US Children in the Post-Rotavirus Vaccine Era. *J Pediatric Infect Dis Soc* **2019**, *8*, 414–421, doi:10.1093/jpids/piy077.
63. Honjo, S.; Kuronuma, K.; Fujiya, Y.; Nakae, M.; Ukae, S.; Nihira, H.; Yamamoto, M.; Akane, Y.; Kondo, K.; Takahashi, S.; et al. Genotypes and Transmission Routes of Noroviruses Causing Sporadic Acute Gastroenteritis Among Adults and Children, Japan, 2015-2019. *Infect Genet Evol* **2022**, *104*, 105348, doi:10.1016/j.meegid.2022.105348.

64. John, J.L.; Mori, D.; Amit, L.N.; Mosiun, A.K.; Chin, A.Z.; Ahmed, K. High Proportion of Norovirus Infection and Predominance of GII.3 [P12] Genotype Among the Children Younger than 5 in Sabah, Malaysian Borneo. *J Clin Virol* **2021**, *143*, 104968, doi:10.1016/j.jcv.2021.104968.
65. Kabue, J.P.; Khumela, R.; Meader, E.; Baroni de Moraes, M.T.; Traore, A.N.; Potgieter, N. Norovirus-Associated Gastroenteritis Vesikari Score and Pre-Existing Salivary IgA in Young Children from Rural South Africa. *Viruses* **2023**, *15*, doi:10.3390/v15112185.
66. Kambhampati, A.K.; Vargas, B.; Mushtaq, M.; Browne, H.; Grytdal, S.; Atmar, R.L.; Vinje, J.; Parashar, U.D.; Lopman, B.; Hall, A.J.; et al. Active Surveillance for Norovirus in a US Veterans Affairs Patient Population, Houston, Texas, 2015-2016. *Open Forum Infectious Diseases* **2019**, *6*, doi:https://dx.doi.org/10.1093/ofid/ofz115.
67. Kébé , O.; Fernandez-Garcia, M.-D.; Zinsou, B.-E.; Diop, A.; Fall, A.; Ndiaye, N.; Vinjé, J.; Ndiaye, K. Prevalence and Genetic Characterization of Noroviruses in Children with Acute Gastroenteritis in Senegal, 2007-2010. *J Med Virol* **2022**, *94*, 2640–2644, doi:10.1002/jmv.27496.
68. Kébé, O.; Ba, B.S.; Thiaw, F.D.; Fall, C.; Barry, M.A.; Boussiengui, L.G.; Diallo, J.P.; CoAg "diarrhées sanglantes" Investigators; Ndiaye, Nd.; Mamadou Ndiaye, E.H.; et al. A Sixteen-Month Monitoring of Human Enteric Viruses Associated with Acute Gastroenteritis in Senegal. *Gut Pathog* **2026**, *18*, 32, doi:10.1186/s13099-026-00806-8.
69. Khamrin, P.; Kumthip, K.; Yodmeeklin, A.; Jampanil, N.; Phengma, P.; Yamsakul, P.; Okitsu, S.; Kobayashi, T.; Ushijima, H.; Maneekarn, N. Changing Predominance of Norovirus Recombinant Strains GII.2[P16] to GII.4[P16] and GII.4[P31] in Thailand, 2017 to 2018. *Microbiol Spectr* **2022**, *10*, e0044822, doi:10.1128/spectrum.00448-22.
70. Khamrin, P.; Kumthip, K.; Yodmeeklin, A.; Okitsu, S.; Motomura, K.; Sato, S.; Ushijima, H.; Maneekarn, N. Genetic Recombination and Genotype Diversity of Norovirus GI in Children with Acute Gastroenteritis in Thailand, 2015-2021. *J Infect Public Health* **2024**, *17*, 379–385, doi:10.1016/j.jiph.2024.01.009.
71. Kittigul, L.; Pairoh, T.; Rupprom, K.; Thongpanich, Y.; Siri, S. Emergence of Norovirus GII.17[P16] in Adult Patients with Acute Gastroenteritis in Thailand during 2021-2023. *PLoS One* **2025**, *20*, e0337513, doi:10.1371/journal.pone.0337513.
72. Kõivumägi, K.; Geller, J.; Toompere, K.; Soeorg, H.; Kallas, E.; Jõgeda, E.-L.; Huik, K.; Lutsar, I. Norovirus Strains Among Children Aged 0-18 Years Hospitalized with Acute Gastroenteritis in Estonia 2015-2016. *J Med Virol* **2022**, *94*, 2632–2639, doi:10.1002/jmv.27495.
73. Lanata, C.F.; Soto, G.; Gil, A.I.; Neyra, J.; Cornejo, R.; Romero, C.; Ecker, L.; Huaylinos, M.L.; La Rosa, S.; Goios, A.; et al. Epidemiology of Norovirus Disease in the First 2 Years of Life: A Prospective Multisite Cohort Study in Lima, Peru. *Int J Infect Dis* **2025**, *150*, 107308, doi:10.1016/j.ijid.2024.107308.
74. Lee, M.; Cho, S.R.; Jo, Y.; Lee, D.Y.; Han, M.G.; Park, S.W. Trends in Norovirus Genotypes in South Korea, 2019-2024: Insights from Nationwide Dual Typing Surveillance. *Viruses* **2025**, *17*, doi:10.3390/v17121572.
75. Li, J.; Zhang, L.; Zou, W.; Yang, Z.; Zhan, J.; Cheng, J. Epidemiology and Genetic Diversity of Norovirus GII Genogroups Among Children in Hubei, China, 2017-2019. *Virol Sin* **2023**, *38*, 351–362, doi:10.1016/j.virs.2023.04.002.
76. Lo, M.; Doan, Y.H.; Mitra, S.; Saha, R.; Miyoshi, S.I.; Kitahara, K.; Dutta, S.; Oka, T.; Chawla-Sarkar, M. Comprehensive Full Genome Analysis of Norovirus Strains from Eastern India, 2017-2021. *Gut Pathogens* **2024**, *16(1) (no pagination)*, doi:https://dx.doi.org/10.1186/s13099-023-00594-5.
77. Lu, L.; Ao, Y.; Jia, R.; Zhong, H.; Liu, P.; Xu, M.; Su, L.; Cao, L.; Xu, J. Changing Predominance of Norovirus Strains in Children with Acute Gastroenteritis in Shanghai, 2018-2021. *Virol Sin* **2023**, *38*, 671–679, doi:10.1016/j.virs.2023.08.005.
78. Lu, L.-J.; Zhong, H.-Q.; Jia, R.; Xu, M.-H.; Liu, P.-C.; Zhu, X.-H.; Xu, J. Resurgence and Predominance of GII.17[P17] Norovirus in Children with Sporadic Acute Gastroenteritis in Shanghai, China. *J Med Virol* **2025**, *97*, e70729, doi:10.1002/jmv.70729.
79. Luchs, A.; Adiwardana, N.S.; Rocha, L.C.D.; Viana, E.; Guadagnucci, S.; Parise, A.; Silva, V.C.M.; Azevedo, L.S.D.; Guiducci, R.; Franca, Y.; et al. Concurrent Circulation of Viral Agents in Pediatric Patients Presenting with

Respiratory Illness and Diarrheal Symptoms in Metropolitan Region of Sao Paulo, Brazil, 2021. *Viruses* **2025**, *17(4) (no pagination)*.
80. Ly, L.T.K.; Doan, Y.H.; Mai, C.T.N.; Takemae, N.; Kageyama, T.; Oka, T.; Parashar, U.D.; Tate, J.E.; Trang, N.V. Clinical, Molecular and Evolutionary Characterization of Noroviruses in Hospitalized Children with Acute Gastroenteritis in Vietnam, 2016 to 2021. *J Med Virol* **2026**, *98*, e70876, doi:10.1002/jmv.70876.
81. Ma, J.; Chen, Q.; Yuan, F.; Cao, M.; Gao, J.; Yang, C.; Tan, M.; Xian, R.; Gao, L.; Kuai, W. Prevalence and Genotype Distribution of Norovirus in Ningxia Hui Autonomous Region, China, from 2011 to 2022. *Virol J* 2024, *21*, 232.
82. Ofosu-Appiah, L.H.; Negoro, M.; Amexo, J.X.; Amelor, D.K.; Tonto, P.B.; Laryea, D.O.; Yamasaki, K.; Asiedu-Bekoe, F.; Sugata, K.; Hori, H.; et al. Clinical Impact and Genetic Analysis of Enteric Viruses Associated with Acute Gastroenteritis in Greater Accra, Ghana: A Comprehensive Study of Five Viruses. *J Med Virol* 2025, *97*, e70216.
83. Ortiz-Quintero, J.; Cabrera, Y.; Bourdett-Stanziola, L.; Ferrera, A. Molecular Characterization of Enteric Viruses Causing Acute Gastroenteritis Among Children under 5 Years Old in Distrito Central, Honduras. *Intervirology* **2024**, *67*, 83–98.
84. Ozawa, K.; Oka, T.; Takeda, N.; Hansman, G.S. Norovirus Infections in Symptomatic and Asymptomatic Food Handlers in Japan. *J Clin Microbiol* **2007**, *45*, 3996–4005, doi:10.1128/JCM.01516-07.
85. Payne, D.C.; Vinjé, J.; Szilagyi, P.G.; Edwards, K.M.; Staat, M.A.; Weinberg, G.A.; Hall, C.B.; Chappell, J.; Bernstein, D.I.; Curns, A.T.; et al. Norovirus and Medically Attended Gastroenteritis in U.S. Children. *N Engl J Med* **2013**, *368*, 1121–1130, doi:10.1056/NEJMsa1206589.
86. Pham, N.T.K.; Khamrin, P.; Shimizu-Onda, Y.; Hoque, S.A.; Trinh, Q.D.; Komine-Aizawa, S.; Okitsu, S.; Maneekarn, N.; Hayakawa, S.; Yoshimune, K.; et al. Genetic Diversity and Declining Norovirus Prevalence in Infants and Children during Japan's COVID-19 Pandemic: A Three-Year Molecular Surveillance. *Arch Virol* **2023**, *168*, 231, doi:10.1007/s00705-023-05856-w.
87. Phengma, P.; Khamrin, P.; Jampanil, N.; Yodmeeklin, A.; Ukarapol, N.; Maneekarn, N.; Kumthip, K. Molecular Epidemiology and Characterization of Norovirus and Sapovirus in Pediatric Patients with Acute Diarrhea in Thailand, 2019-2020. *J Infect Public Health* **2022**, *15*, 1013–1019, doi:10.1016/j.jiph.2022.08.006.
88. Pimenta, Y.C.; Moreira, G.M.S.; da Silva Souza, W.; Olivares, A.I.O.; Svensson, L.; Leite, J.P.G.; Nordgren, J.; de Moraes, M.T.B. Epidemiological Profiles of Norovirus Genotypes in Children with and without Acute Gastroenteritis from the Northwestern Amazon Region. *Infect Genet Evol* **2025**, *134*, 105804, doi:10.1016/j.meegid.2025.105804.
89. Pitkänen, O.; Markkula, J.; Hemming-Harlo, M. A Major Decrease in Viral Acute Gastroenteritis in Hospitalized Finnish Children as Rotavirus Returns as the Most Detected Pathogen. *Int J Infect Dis* **2022**, *114*, 273–278, doi:10.1016/j.ijid.2021.11.016.
90. Pitkänen, O.; Markkula, J.; Hemming-Harlo, M. Sapovirus, Norovirus and Rotavirus Detections in Stool Samples of Hospitalized Finnish Children with and without Acute Gastroenteritis. *Pediatr Infect Dis J* **2022**, *41*, e203–e207, doi:10.1097/inf.0000000000003493.
91. Reyes, Y.; González, F.; Gutiérrez, L.; Blandón, P.; Centeno, E.; Zepeda, O.; Toval-Ruíz, C.; Lindesmith, L.C.; Baric, R.S.; Vielot, N.; et al. Secretor Status Strongly Influences the Incidence of Symptomatic Norovirus Infection in a Genotype-Dependent Manner in a Nicaraguan Birth Cohort. *J Infect Dis* **2022**, *225*, 105–115, doi:10.1093/infdis/jiab316.
92. Sai, L.; Wang, G.; Shao, L.; Liu, H.; Zhang, Y.; Qu, C.; Ma, L. Clinical and Molecular Epidemiology of Norovirus Infection in Adults with Acute Gastroenteritis in Ji'nan, China. *Arch Virol* **2013**, *158*, 2315–2322, doi:10.1007/s00705-013-1753-0.
93. Sakon, N.; Yamazaki, K.; Nakata, K.; Kanbayashi, D.; Yoda, T.; Mantani, M.; Kase, T.; Takahashi, K.; Komano, J. Impact of Genotype-Specific Herd Immunity on the Circulatory Dynamism of Norovirus: A 10-Year Longitudinal Study of Viral Acute Gastroenteritis. *J Infect Dis* **2015**, *211*, 879–888, doi:10.1093/infdis/jiu496.

94. Sharif, N.; Ahmed, S.N.; Sharif, N.; Alzahrani, K.J.; Alsuwat, M.A.; Alzahrani, F.M.; Khandaker, S.; Monifa, N.H.; Okitsu, S.; Parvez, A.K.; et al. High Prevalence of Norovirus GII.4 Sydney Among Children with Acute Gastroenteritis in Bangladesh, 2018-2021. *J Infect Public Health* **2023**, *16*, 1015–1022, doi:10.1016/j.jiph.2023.05.002.
95. Supadej, K.; Khamrin, P.; Kumthip, K.; Malasao, R.; Chaimongkol, N.; Saito, M.; Oshitani, H.; Ushijima, H.; Maneekarn, N. Distribution of Norovirus and Sapovirus Genotypes with Emergence of NoV GII.P16/GII.2 Recombinant Strains in Chiang Mai, Thailand. *J Med Virol* **2019**, *91*, 215–224, doi:10.1002/jmv.25261.
96. Tang, M.B.; Chen, C.H.; Chen, S.C.; Chou, Y.C.; Yu, C.P. Epidemiological and Molecular Analysis of Human Norovirus Infections in Taiwan during 2011 and 2012. *BMC Infect Dis* **2013**, *13*, 338, doi:10.1186/1471-2334-13-338.
97. Tegegne, D.; Gelaw, A.; Alemayehu, D.H.; Seyoum, T.; Leta, D.; Ferede, G.; Mulu, A.; Gelaw, B. Genetic Diversity and Distribution of Noroviruses Among All Age Groups of Patients with Diarrhea in Amhara National Regional State, Ethiopia. *PLoS One* 2024, *19*, e0303887.
98. Umair, M.; Jamal, Z.; Haider, S.A.; Javed, H.; Hakim, R.; Ali, Q.; Usman, M.; Suleman, R.; Saeed, M.U.; Salman, M. Molecular Epidemiology and Genomic Insights into Norovirus and Coinfections: A 2023 Tertiary Care Hospital Study on Pediatric Acute Gastroenteritis in Pakistan. *Pediatr Infect Dis J* **2025**, *44*, 1225–1230, doi:10.1097/inf.0000000000004915.
99. Wang, X.; Wei, Z.; Guo, J.; Cai, J.; Chang, H.; Ge, Y.; Zeng, M. Norovirus Activity and Genotypes in Sporadic Acute Diarrhea in Children in Shanghai During 2014-2018. *Pediatr Infect Dis J* **2019**, *38*, 1085–1089, doi:10.1097/inf.0000000000002456.
100. Wang, X.; Liu, W.; Hu, M.; Ma, H.; He, Y.; Wang, B.; Li, K.; Zhang, R.; Fu, J.; Zhang, H.; et al. Genetic Diversity of Norovirus in Shenzhen Based on Continuous Surveillance from 2016 to 2022. *Front Cell Infect Microbiol* **2025**, *15*, 1593610, doi:10.3389/fcimb.2025.1593610.
101. Wu, X.; Han, J.; Chen, L.; Xu, D.; Shen, Y.; Zha, Y.; Zhu, X.; Ji, L. Prevalence and Genetic Diversity of Noroviruses in Adults with Acute Gastroenteritis in Huzhou, China, 2013-2014. *Arch Virol* **2015**, *160*, 1705–1713, doi:10.1007/s00705-015-2440-0.
102. Yan, G.J.H.; Hewitt, J.; Mercer, L.K.; Harding, E.F.; Croucher, D.; Russo, A.G.; Huntington, P.G.; Mackenzie, J.M.; Rawlinson, W.D.; White, P.A. Molecular Epidemiology and Evolution of Norovirus in Australia and New Zealand, 2018 to 2020. *Infect Dis (Lond)* **2025**, *57*, 766–781, doi:10.1080/23744235.2025.2479133.
103. Yi, J.; Wahl, K.; Sederdahl, B.K.; Jerris, R.R.; Kraft, C.S.; McCracken, C.; Gillespie, S.; Anderson, E.J.; Kirby, A.E.; Shane, A.L.; et al. Molecular Epidemiology of Norovirus in Children and the Elderly in Atlanta, Georgia, United States. *J Med Virol* **2016**, *88*, 961–970, doi:10.1002/jmv.24436.
104. Yu, F.; Feng, X.; Ye, H.; Cai, Q.; Jiang, J.; Zhang, T.; Pan, F.; Weng, W. The Increased Detection of Recombinant Norovirus GII.4 Sydney_2012[P16] Strains in Hospitalized Pediatric Patients with Gastroenteritis in Shanghai, China. *BMC Infectious Diseases* **2026**, *26(1) (no pagination)*, doi:10.1186/s12879-026-12565-7.
105. Zheng, W.; Mu, X.; Wang, S.; Qiu, T.; Wang, X. Increase of GII.17 Norovirus Infections During the 2024-2025 Season in Multiple Cities, China. *J Med Virol* **2026**, *98*, e70858, doi:10.1002/jmv.70858.
106. Zhu, X.; Zhang, P.; Yan, W.; Dong, F.; Xu, D.; Yuan, R.; Ji, L. A New Wave of Resurgence for GII.4 Sydney in Huzhou, Particularly GII.4 Sydney[P16], between 2019 and 2023. *BMC Infect Dis* 2025, *25*, 241.
